# Pediatric Blastic Plasmacytoid Dendritic Cell Neoplasm, Clinical Features and Immunophenotype: A Case Report

**DOI:** 10.7759/cureus.34549

**Published:** 2023-02-02

**Authors:** Amaranto Suárez, Nathalie Soler, Alejandra Calderon, Bibiana Martinez, Martha Piña

**Affiliations:** 1 Pediatric Oncology, Instituto Nacional de Cancerología, Bogotá, COL; 2 Pediatric Oncology, Instituto Nacional de Cancerología, Bogota, COL; 3 Bacteriologist, specialising in Haematology, Instituto Nacional de Cancerología, Bogota, COL

**Keywords:** morphology, clinics, leukemia, pediatric, blastic plasmacytoid dendritic cell neoplasm

## Abstract

Blastic plasmacytoid dendritic cell neoplasm (BPDCN) is a rare but aggressive malignancy with high mortality involving the skin and hematopoietic system. Clinical suspicion is difficult, and management of skin lesions is challenging due to their indolent course prior to dissemination. We describe a patient with isolated skin involvement who progressed to CD4+/CD56+ and CD123+ acute leukemia.

## Introduction

Blastic plasmacytoid dendritic cell neoplasm (BPDCN) is a highly aggressive hematological malignancy that accounts for less than 1% of acute leukemias and 0.7% of cutaneous lymphomas. It primarily involves the skin (64%-100%) and bone marrow (60%-90%), with secondary involvement of lymph nodes (40%-50%), soft tissues and the central nervous system. It is derived from a subset of precursor cells called plasmacytoid dendritic cells or type II dendritic cells, characterized by the expression of CD123, CD4, and CD56 in the absence of markers of other lineages [1,2]. Although the disease predominantly affects older adults, with a median age of 65 years, it has been reported in other age groups, including infants and children [3-5].

The first reported case was published in 1994 by Adachi et al. [6] who described a CD4+ lymphoma with high CD56 expression; subsequently, these neoplasms have been given various names, including natural killer cell (NK cell) lymphoma and CD4+/CD56+ hematodermic neoplasm [7]. Although the expression of CD4 and CD56 suggests a T or NK lymphocytic lineage, the presence of CD123+ and TCL1+ leads BPDCN to be currently considered a type II plasmacytoid dendritic cell-derived neoplasm [7] and to be recognized as a distinct entity by the World Health Organization (WHO) since 2008 [3,7,8]. The disease is characterized by its clinical aggressiveness with a tendency to recur over time and high mortality, in most cases within the first year of diagnosis [9,10].

Experience in pediatric patients with BPDCN is very limited, with few reported cases and small case series described in the literature. In a systematic review published in 2017 by Kim et al. [5], only 74 of 357 cases, were children. We report a case of a patient with blastic plasmacytoid dendritic cell leukemia to educate the medical community about the clinical and diagnostic features of this extremely rare neoplasm in children and to add another case of BPDCN to the published series.

## Case presentation

A four-year-old female was referred to our center with a six-month history of the appearance of nodular, circumscribed, multiple, sometimes confluent, violaceous lesions on the skin. It began with a single lesion on the lateral aspect of the right leg that was interpreted as an infected hematoma. She received courses of antibiotic therapy without resolution of symptoms; she was therefore taken for surgical drainage but without intraoperative findings of hemorrhagic or infectious debris. Subsequently, lesions with similar characteristics appeared on different parts of the body: face, trunk, and extremities. She was seen three more times for the same skin lesions and was given antibiotics without improvement; on her last visit, a physical examination revealed lymphadenopathy in the neck, supraclavicular region, hepatomegaly, and splenomegaly. A complete blood count (CBC) showed anemia and thrombocytopenia. Dengue was ruled out.

**Figure 1:**
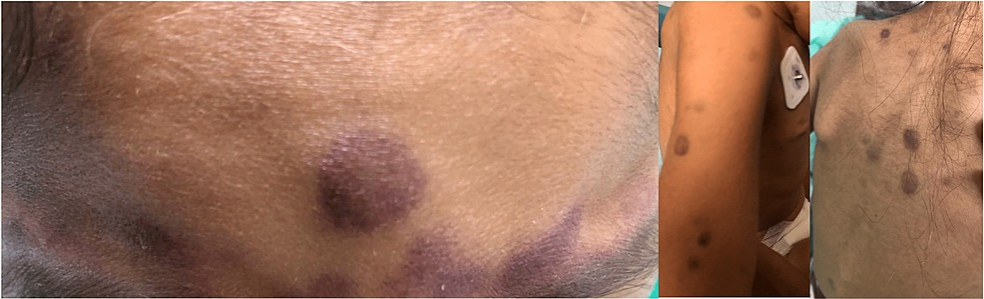
Nodular skin lesions and circular violaceous papules with regular margins.

On admission, the patient presented with numerous violaceous cutaneous lesions, papules, and nodules > 2 cm in size with regular, well-defined circumferential borders; these lesions were located on the face, neck, upper chest, back, and upper and lower limbs, several were tender on palpation (Figure 1).

The admission CBC showed hemoglobin 8.0 g/dL, leucocytes 10,310/mm^3^, neutrophils 2,340/mm^3^ and platelets 105,000/mm^3^. Peripheral blood smear showed 76% blasts. Blood chemistry for liver and kidney function was normal.

**Figure 2:**
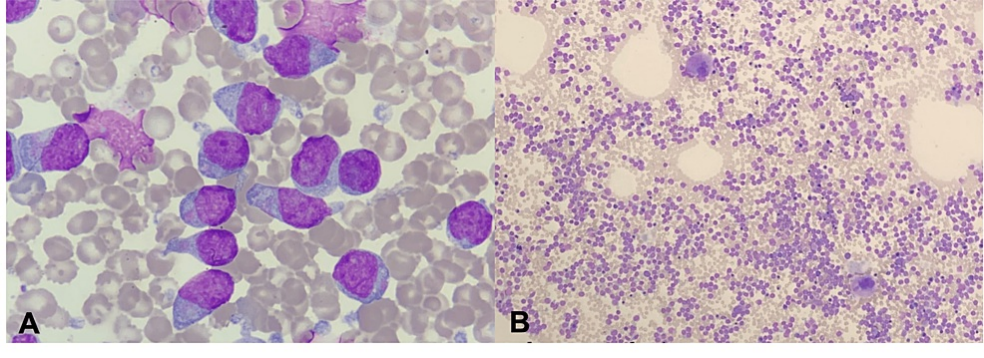
(A) Wright's stain 100x. Bone marrow aspirate showing medium-sized blasts with moderate amounts of greyish cytoplasm, pseudopodia, lax chromatin and nucleoli. (B) Wright's stain 10x. Bone marrow aspirate infiltrated by a monomorphic population.

Bone marrow examination revealed hypercellularity with a 91% population of large blasts with moderate amounts of greyish cytoplasm, pseudopodia, lax chromatin and nucleoli. Minimal residual hematopoiesis was observed (Figures 2A, 2B).

**Figure 3:**
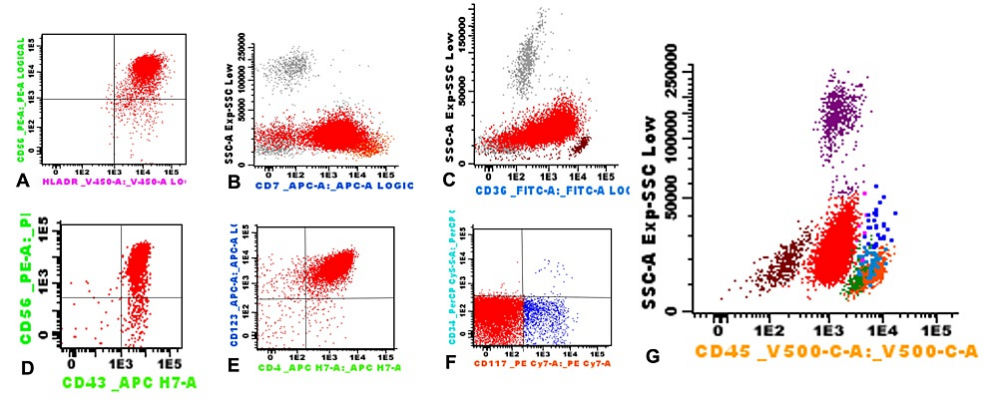
Bone marrow flow cytometry showed the presence of blastic plasmacytoid dendritic cells (red events) with expression of CD56 (A, D), HLA DR (A), CD7(B), CD36 (C), CD43 (D), CD4 (E), CD123(E), partial weak CD117 expresión (F), weak CD45 expresión (G). Courtesy of Flow Cytometry Laboratory.

Immunophenotyping by flow cytometry showed the presence of blastic plasmacytoid dendritic cells with expression of CD56, HLA DR, CD43, CD4, CD123, CD7, CD36, weak CD45 expression, partial weak CD117 expression and absence of b/T lineage cell markers (Figures 3A-3G). Cerebrospinal fluid cytology was negative.

**Figure 4:**
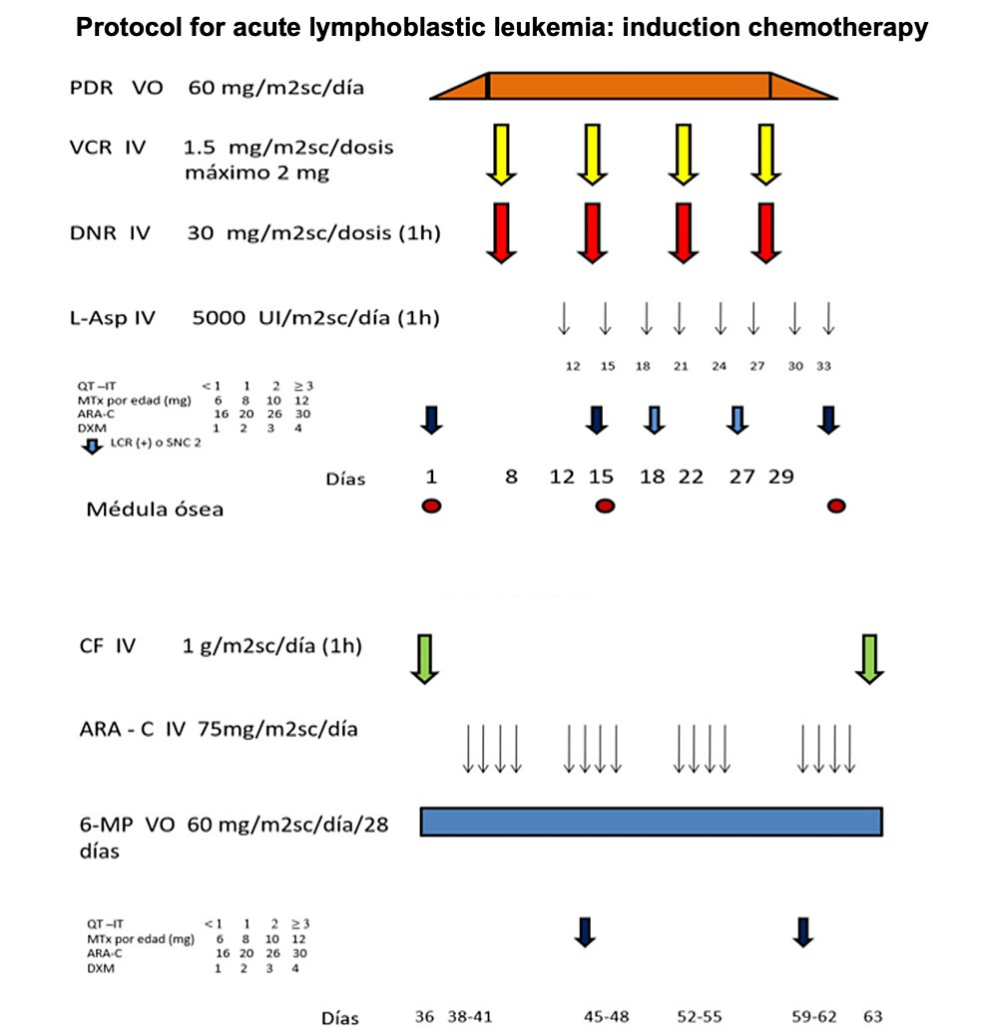
Protocol for acute lymphoblastic leukemia: induction chemotherapy

Chemotherapy was started according to the institutional protocol for acute lymphoblastic leukemia (ALL) based on the BFM-ALLIC 2009 strategy (ALL-INC 2012). She received induction chemotherapy (Figure 4).

**Figure 5:**
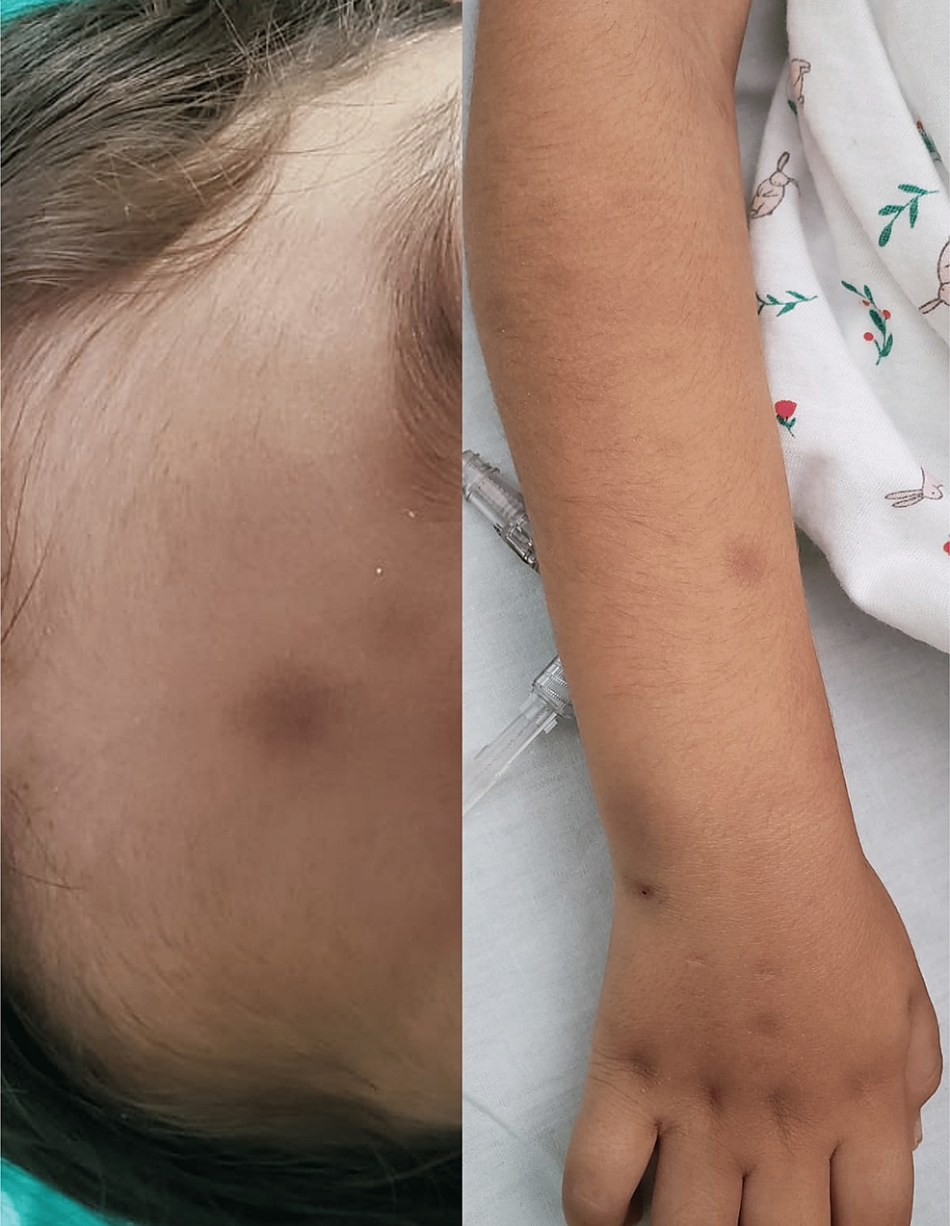
Nodular skin lesions after induction phase chemotherapy. Single flat lesions may be seen, with decreased size and discolouration.

Evaluation on day 15 of induction showed 1.6% blasts by morphology and 1% by flow cytometry. Evaluation at the end of induction showed 0.3% abnormal dendritic cells by morphology and 0.17% by flow cytometry with an expression of CD123, partial expression of CD56 and CD7, weak CD4, weak HLA DR and weak CD45. Physical examination revealed resolution of most skin lesions and a significant decrease in the largest lesions at diagnosis (Figure 5).

She continued treatment with the consolidation phase. At the end of this phase (day 76), bone marrow evaluation showed 1.3% blasts by morphology and 1.1% by flow cytometry with expression of CD13, CD33, CD117, CD34, HLA DR and CD45.

The patient was evaluated by the pediatric hematopoietic stem cell transplantation (HSCT) group. Histocompatibility testing was ordered. During the next phase of chemotherapy, she developed febrile neutropenia and septic shock requiring admission to intensive care where she died.

## Discussion

BPDCNs are rare hematolymphoid neoplasms that often present with skin lesions followed by rapid leukemic dissemination [9]. BPDCN is considered a hematodermal neoplasm because of its strong tendency to debut with cutaneous involvement. These lesions vary from hematoma-like macules to purplish plaques or nodules; focal lesions often affect the head and lower extremities, although they may spread throughout the body [11,12]. As shown in Figure 1, the characteristic lesions of the cutaneous involvement are non-specific and are easily confused with other differential diagnoses [3,4,13].

In a systematic review of the literature on BPDCN published in 2017, Kim et al. [5] reported other common sites of involvement in children, including bone marrow (66%), peripheral blood (40%), lymph nodes (46%), liver (16%), spleen (26%), and central nervous system (47%). The clinical course of the case showed the natural history of a disease that began with skin involvement and was not initially suspected to be malignant, presenting after six months with lymphadenopathy, visceromegaly, and a hemogram showing anemia, thrombocytopenia and blasts in the peripheral blood. Hematological features in our case are consistent with those reported by Feuillard et al. [14], where the most common finding was thrombocytopenia in 78% of cases, anemia in 65%, leukopenia with neutropenia in 34% and leukocytosis in 22% [14,15].

Morphologically, BPDCNs are characterized by a monomorphic infiltrate of medium-sized blast cells resembling lymphoblasts or myeloblasts. The nucleus is irregular with fine chromatin and some nucleoli present, and the cytoplasm is usually sparse, agranular, and greyish in color [8]. 

Immunophenotypic features for the diagnosis of BPDCN can be identified by immunohistochemistry or flow cytometry. The classic profile is an expression of CD4, CD56, CD45RA, and HLA-DR (human leukocyte antigen isotype DR) and the plasmacytoid dendritic cell-associated antigens (pDC) CD123 (interleukin-3 receptor), CTL-1 (T-cell leukemia/lymphoma 1), BDCA-2/CD303, CD304, CD2AP; and lacks expression of CD3, CD13, CD14, CD16, CD19, CD20, CD34, myeloperoxidase and lysozyme [11,12,16]. Although it has been reported that approximately 8% of cases may be CD4 and CD56 negative, this does not exclude the diagnosis if other plasmacytoid dendritic cell-associated antigens (CD123, TCL1, or CD303) are expressed [8].

Both the morphology and immunophenotype by flow cytometry of the patient's bone marrow are consistent with those described for the diagnosis of blastic plasmacytoid dendritic cell leukemia (Figures 2, 3). The fourth revised edition of the WHO classification of hematopoietic tumors [8] states that CD4, CD45, CD56, and CD123 positivity is considered a pathognomonic phenotype for BPDCN in the absence of antigens associated with a specific myeloid lineage (CD13, CD33, and myeloperoxidase), T cells (CD3, CD5) and B cells (CD19, CD20) [8,17].

A variety of treatments have been used in adult patients, including chemotherapy regimens based on protocols for non-Hodgkin's lymphoma, myeloid leukemia, and ALL, with and without HSCT. In addition, the low incidence of the disease makes it difficult to define an optimal therapeutic approach; however, in children, treatment with high-risk ALL protocols is the most commonly used strategy and is associated with the best-reported outcomes [4,5,13].

In adults, stem cell transplantation after remission is considered part of the standard of care for BPDCN. Jegalian et al. [3] reported that pediatric patients who underwent HSCT had an overall survival of 67%, while the overall survival of children who did not undergo transplantation was 74%; although the number of cases is small, the authors concluded that the role of HSCT in pediatric cases is unclear. They recommend that treatment for children with BPDCN should include high-risk ALL therapy with central nervous system prophylaxis and reserve transplantation for patients in second complete remission or in cases where initial treatment does not induce a complete remission [3,4].

Recently, the Food and Drug Administration (FDA) approved the use of a cytotoxin targeting CD123-expressing cells (SL-401), known as tagraxofusp, for the treatment of BPDCN in both adults and children over two years of age. Tagraxofusp is a fusion protein of human IL3 and truncated diphtheria toxin produced in E. coli by recombinant DNA technology [18,19]. Sun et al. [20] reported the first three cases treated with Tagraxofusp with a good safety profile and minimal toxicity. One patient with relapsed BPDCN treated with an ALL regimen did not respond to tagraxofusp. The other two cases showed a partial response with clinical improvement after two cycles of treatment; however, the response was transient and both patients showed disease progression after two to three months of treatment [20].

Our patient received induction treatment with a high-risk ALL protocol and showed a partial response in the bone marrow at the end of induction with 0.3% abnormal dendritic cells and 0.17% by flow cytometry with CD123 expression, accompanied by a significant improvement in skin lesions (Figure 5). After consolidation chemotherapy and awaiting HSCT, the patient developed febrile neutropenia with septic shock and died.

## Conclusions

There is a need to increase awareness and sensitivity of the clinical suspicion of BPDCNs among healthcare providers treating children and adolescents. A better understanding of the genetic and molecular mechanisms will lead to the definition of prognostic markers to guide risk-adapted treatment.
